# More than just “vaginal dryness”: sexual dysfunction correlates with genitourinary anatomy changes in female cancer survivors

**DOI:** 10.1007/s00520-025-10046-2

**Published:** 2025-11-13

**Authors:** Erin Kobiella, Sanjana Satish, Fay Pon, Lia Jueng, Chloe Shields, Melissa Curran, Tizeta Wolde, Jessica F. Moore, Samantha Greenseid, Tulay Koru-Sengul, Wei Zhao, Frank Penedo, Kristin E Rojas

**Affiliations:** 1 Medical Graduate Education, University of Miami Miller Medical School, Miami, FL USA; 2 Department of Obstetrics and Gynecology, University of Pennsylvania Perelman School of Medicine, Philadelphia, PA USA; 3 Department of Obstetrics, Gynecology, and Reproductive Sciences, University of Miami Miller School of Medicine, 1475 NW 12 Ave, Miami, FL 33136 USA; 4 Department of Public Health Sciences, University of Miami Miller School of Medicine, Miami, FLFL USA; 5 Sylvester Comprehensive Cancer Center, University of Miami Miller School of Medicine, 1475 NW 12Th Ave, Miami, FL 33136 USA; 6 Department of Psychology, University of Miami Miller School of Medicine, Miami, FL USA; 7 Department of Medicine, University of Miami Miller School of Medicine, Miami, FL USA; 8 Dewitt-Daughtry Department of Surgery, University of Miami Miller School of Medicine, 1475 NW 12Th Ave, Miami, FL 33136 USA

**Keywords:** Cancer, Sexual dysfunction, Genitourinary syndrome of menopause, Menopause, Survivorship

## Abstract

**Purpose:**

To correlate genitourinary exam (GU) findings to patient-reported sexual dysfunction in female cancer survivors.

**Methods:**

This retrospective cohort study included female cancer patients seen at a South Florida sexual health after cancer program. GU anatomy abnormalities and patient-reported sexual dysfunction were evaluated by Adapted Vulvovaginal Exam Score (AVES) and the Female Sexual Function Index (FSFI), respectively. Multivariate analyses compared, FSFI scores between patients with AVES > 3 vs. 0–3, (AVES > 3 corresponds to more abnormal GU exam). Adjusted odds ratio (aOR) and 95% confidence intervals (95% CI) were calculated.

**Results:**

AVES was calculated for 162 female patients treated between 2020–2022. Median age was 46; 57% were Hispanic, and 79% had breast cancer. Common symptoms included vaginal dryness (55%) and dyspareunia (45%). Of 108 women with FSFI scores, 97% met criteria for female sexual dysfunction (FSD). 23% were found to have vaginal stenosis, and 42% had a narrowed vaginal introitus. Those with AVES > 3 had significantly lower FSFI lubrication, orgasm, satisfaction, and pain domain scores. Any endocrine therapy use was associated with worse AVES scores (aOR 0.20, 95% CI 0.05–0.80, p = 0.024), an association strongest with aromatase inhibitor (AIs) use. Low satisfaction scores < 3.6 were nearly three times more likely to have abnormal GU exams (aOR = 2.81; 95% CI: 1.03–7.65; p = 0.044).

**Conclusion:**

FSD in female cancer survivors is associated with previously unreported GU exam changes that can limit or prevent sexual activity through pain and worsened sexual satisfaction. Ongoing work evaluates targeted interventions to improve symptoms and quality of life for this growing survivor population.

**Supplementary Information:**

The online version contains supplementary material available at 10.1007/s00520-025-10046-2.

## Introduction

As a result of advancements in targeted therapies, the number of cancer survivors in the United States is expected to exceed 20 million by 2026 with approximately 10.3 million survivors predicted to be women [1]. Adding to the challenges that survivors face, many cite sexuality as a major concern and unmet need [1, 2].

Female sexual dysfunction (FSD) is reported in women with all cancer types, including breast, gynecologic, hematologic, colorectal, and head and neck cancers [1–3]. Women with cancer are estimated to have a 2.7- to 3.5-fold increased risk of developing sexual dysfunction compared to women without cancer [3]. In patients with breast cancer, the prevalence of FSD is estimated to be 50–75% [2, 4]. Additionally, an estimated 43% of gynecologic cancer survivors report sexual dysfunction [5], whereas patients with cervical cancer experience sexual dysfunction at an even higher rate of 80% [6]. Sexual difficulties also occur in up to 75% of female patients who are treated for colorectal cancer [7], and 85% of female patients with anal cancer report symptoms of sexual dysfunction, particularly dyspareunia in up to 65% of these patients [8]. Those with an elevated risk for malignancy due to genetic predisposition syndromes also report high rates of negative sexual side effects after risk-reducing surgeries [9], and iatrogenic estrogen suppression related to preventive therapies can incite or worsen menopausal symptoms. Lastly, those with hematologic malignancies have an increased risk of immune-mediated vulvar disorders such as vulvovaginal graft-versus-host disease (GVHD) and lichen sclerosis [10]. Negative changes to female sexual health can be the sequelae of any type of malignancy, not just breast and gynecologic types. Despite the high rates of distressing sexual symptoms in female cancer survivors, sexual dysfunction remains critically undertreated.

Most commonly, female cancer survivors experience treatment-induced genitourinary syndrome of menopause (GSM), which can include symptoms of vaginal dryness, dyspareunia, diminished libido, poor arousal, and difficulties with orgasm. Chemotherapy, pelvic radiation, bilateral salpingo-oophorectomy (BSO), and estrogen suppression increase the risk of GSM [11], and premenopausal patients who receive chemotherapy and experience premature iatrogenic menopause may be unprepared for these abrupt changes that impact their quality of life [12]. Although genitourinary exams are recommended for cancer survivors who present for evaluation of sexual concerns, few studies detail the anatomic disruptions encountered during the exams of female survivors and the effect such changes have on the patient’s sexual well-being and overall quality of life. Therefore, the objective of the current study was to describe genitourinary exam findings in a diverse population of female cancer survivors and correlate these findings to patient-reported sexual dysfunction.

## Methods

This retrospective cohort study included female cancer patients presenting to a sexual health after cancer program at an NCI-designated cancer center in South Florida between November 2020 and June 2022. The study was approved by the University of Miami Institutional Review Board, and research was conducted according to the principles of the Declaration of Helsinki. Informed consent was obtained from all individual participants. The MUSIC™ (Menopause, Urogenital, Sexual Health and Intimacy Clinic) Sexual Health After Cancer Program provides a safe space where stigmatized topics such as sexual well-being can be discussed and treated. Patients referred to the MUSIC™ Program receive a one-on-one clinic visit with a subspecialized board-certified women’s health specialist to discuss symptoms related to sexual wellness. Patients also undergo a complete physical exam and receive a personalized treatment plan. All intake paperwork in the Program is available in English and Spanish.

Electronic medical records were queried for demographic data, clinical history, cancer diagnoses and treatment, and patient-reported sexual health symptoms (e.g., vaginal dryness, painful sex, low desire). During the clinical encounter, patients were asked if ability to achieve orgasm during sexual activity was the same, more difficult, or impossible after cancer treatment compared to before diagnosis. Similarly, patients were asked if penetrative sexual activity was possible but uncomfortable, possible but painful, or impossible due to pain after treatment for cancer. This data was collected from clinic charts. Data on genitourinary (GU) exam findings and treatment plan after the first program visit were also collected.

As part of the intake paperwork, Female Sexual Function Index (FSFI) was administered in English and Spanish at the first visit to measure patient-reported sexual function. The FSFI is a 19-item Likert scale questionnaire that assesses female sexual functioning through six domains: desire, arousal, lubrication, orgasm, satisfaction, and pain. The FSFI was validated for use in gynecologic and cervical cancer populations in 2012 [13] and breast cancer populations in 2015 [14]. The maximum FSFI score is 36, and a total FSFI score ≤ 26.0 indicates female sexual dysfunction [15]. Of note, desire domain scores < 5.0 are indicative of hypoactive sexual desire disorder [16]. Total scores and domain scores were calculated.

An Adapted Vulvovaginal Exam Score (AVES) was calculated for each patient who underwent a genitourinary (GU) exam. This score was developed from the Vaginal Health Assessment described in the supplementary material of a previous publication [17] and assesses characteristics of the vagina and vulva, including vaginal rugosity, vascularity, epithelial integrity, elasticity, and moisture. Additionally, the terms epithelial integrity, elasticity, and moisture were interpreted to include an assessment of the vestibule. For each individual exam finding, a score of 0 corresponds to normal findings, a score of 1 indicates mild abnormality, and a score of 2 indicates severe abnormality. Based on the distribution of scores in this population, the AVES cutoff point for severe exam abnormalities was established at the first quartile. Subsequently, AVES 0–3 correlated to a normal GU exam, whereas AVES greater than 3 correlated to severe GU exam abnormalities. The AVES was used for variable comparisons. The presence of vestibular irritation, vulvar atrophy, vulvar irritation, and narrowing of the introitus was also noted and rates of these findings were calculated.

Furthermore, the prevalence of vaginal stenosis in this sample was determined based on the presence of vaginal shortening, vaginal agglutination, and/or vaginal adhesions/scarring on genitourinary exam. Vaginal stenosis was assessed visually by identifying fibrous scar bands and annular strictures on exam. For measurement of vaginal length, a scopette was placed in the apex or posterior fornix of the vagina and the scopette was marked at the level of the hymenal remnant or vestibule. Less than 4 cm was defined as severe vaginal shortening, 4–6 cm was moderate shortening, and > 6 cm was considered normal. These characteristics were pulled from the VHA and measured separately to calculate a rate of vaginal stenosis.

Data was collected and stored using the REDCap (Research Electronic Data Capture) tool. REDCap is a secure, web-based software platform designed to support data capture for research studies, providing 1) an intuitive interface for validated data capture; 2) audit trails for tracking data manipulation and export procedures; 3) automated export procedures for seamless data downloads to common statistical packages; and 4) procedures for data integration and interoperability with external sources [18, 19].

Demographic, clinical, and treatment characteristics and patient-reported symptoms were summarized with descriptive statistics. Chi-square test for association and Fisher’s exact test, if applicable, were employed to correlate AVES groups to patient characteristics. Mean FSFI total and domain scores were compared between AVES groups (0–3 vs. > 3) using student’s t-test. Domain score cutoffs were used based on previously published studies. Multivariable binary logistic regression model was fit for high AVES (> 3) with reference group of low-AVES (0–3) by incorporating clinically relevant variables in the model as covariates. Adjusted odds ratios (aOR) and corresponding 95% confidence intervals along with p-values are calculated. Data management and statistical analysis were performed with SAS version 9.4 for Windows (SAS Institute Inc., Cary, NC).

## Results

### Demographics

Between November 2020 and June 2022, 162 female cis-gender patients were evaluated, and AVES was calculated for 139 patients (86%). Overall, the mean age was 46 years (range: 19–70), and most patients were under 50 years old (63%). Many patients were White (84%) and/or Hispanic (57%). Most patients reported English as their primary language (74%), and a significant proportion of patients reported a primary language of Spanish (26%). One-quarter of the population reported current or previous tobacco use, and one-quarter of patients had Medicare or Medicaid for insurance coverage (27%). Most patients had breast cancer (79%), followed by gynecologic cancer (9%). A small number of patients had a history of gastrointestinal (GI) malignancy (2%), hematologic malignancy (8%), or other cancer subtypes such as lymphoma, renal malignancies, and pseudomyxoma peritonei (1.4%). There were no significant differences in AVES groups (0–3 and > 3) by race, ethnicity, primary language, smoking status, insurance, cancer type or stage, although there was a trend towards a greater proportion of patients ≥ 50 to have more higher AVES score (more abnormal GU exams), as shown in Table 1.

**Table 1:** Demographic and clinical characteristics of the study population in total and by AVES exam group

		Adapted Vulvovaginal Exam Score (AVES)		
		0–3	> 3	
Characteristic	N (%)^a^	N (%)	N (%)	P-value*
Cis-gender female	139 (100)	50 (36)	89 (64)	-
Age at diagnosis > 50 < 50 Mean (SD) Min, Max	50 (36) 87 (63) 46 (11) 19, 70	13 (26) 37 (43) - -	37 (74) 50 (57) - -	0.053
Race White Black/Other	116 (84) 16 (12)	43 (37) 5 (31)	73 (63) 11 (69)	0.650
Ethnicity Hispanic Non-Hispanic	79 (57) 56 (40)	32 (40) 16 (29)	47 (60) 40 (71)	0.154
Primary Language English Spanish	103 (74) 36 (26)	36 (35) 14 (39)	67 (65) 22 (61)	0.672
Smoking Status Never Former/Current Unknown	102 (73) 35 (25) 2 (1)	37 (36) 12 (34) -	65 (64) 23 (66) -	0.832
Insurance Type Private Medicare/Medicaid Other	87 (63) 38 (27) 14 (10)	31 (36) 15 (40) 4 (29)	56 (64) 23 (60) 10 (71)	0.764
Menopausal at Diagnosis Premenopausal Postmenopausal	87 (63) 46 (33)	- 11 (24)	- 35 (76)	0.045
Cancer Type Breast Gynecologic Leukemia Gastrointestinal Other	110 (79) 13 (9) 11 (8) 3 (2) 2 (1)	45 (41) 1 (8) 3 (27) - 1 (50)	65 (59) 12 (92) 8 (73) 3 (100) 1 (50)	0.096
Clinical Tumor Stage DCIS-Stage II Stage III-IV Unknown	67 (48) 18 (13) 54 (39)	29 (43) 5 (28) -	38 (57) 13 (72) -	0.233
Hysterectomy performed^b^	54 (34)	13 (28)	33 (72)	0.193
Bilateral Oophorectomy performed^b^	52 (33)	12 (27)	32 (73)	0.134
Treatment received^c^ Endocrine Therapy Tamoxifen only Tamoxifen + AI or AI only^d^ Chemotherapy Radiation Immunotherapy Surgery^e^ Surgery and Chemotherapy Surgery and Endocrine Therapy Other	93 (67) 21 (22) 72 (78) 103 (74) 86 (62) 18 (13) 124 (89) 89 (64) 32 (23) 9 (6)	39 (42) 14 (67) 25 (35) 33 (32) 29 (34) 7 (39) 46 (37) 29 (33) 16 (50) 2 (22)	54 (58) 7 (33) 47 (65) 70 (68) 57 (66) 11 (61) 78 (63) 60 (67) 16 (50) 7 (78)	0.011 0.102 0.334 0.735 0.427 0.406 0.406 0.406
Type of Surgery Mastectomy Lumpectomy Hysterectomy Other	68 (49) 39 (28) 13 (9) 4 (3)	30 (44) 15 (38) 1 (8) -	38 (56) 24 (62) 12 (92) 4 (100)	0.049

DCIS: Ductal Carcinoma In-Situ; AVES: Adapted Vulvovaginal Exam Score

* P-values are calculated either with chi-square test for association or Fisher’s exact test, excluding unknown values

^a^Some percentages do not add up to 100% due to non-response, unknown or missing values

^b^History of surgery reported at time of first MUSIC visit for ANY reason (not necessarily cancer treatment)

^c^Same patient can be in multiple treatment categories

^d^AI = Aromatase inhibitor (anastrozole, letrozole, exemestane)

^e^Surgery done for cancer treatment purposes

### Presenting symptoms, menopausal status, and genitourinary exam findings

The most common presenting symptoms were vaginal dryness (55%), dyspareunia (45%), low sexual desire (36%), and bothersome hot flashes (22%) (Table 1). At presentation, nearly 1 in 5 women disclosing penetrative sexual activity changes (n = 66) reported that penetration was impossible. Furthermore, of those who reported changes to orgasm (n = 63), 41% disclosed that orgasm was more difficult to achieve, and 18% reported that orgasm was impossible. At the time of cancer diagnosis, most patients were pre-menopausal (63%). Out of all menopausal patients (n = 46), 76% were found to have AVES > 3 (p = 0.045) (Table 2).

**Table 2:** Sexual symptoms and genitourinary exam changes of the study population in total and by AVES group

		Adapted Vulvovaginal Exam Score (AVES)		
		0–3	> 3	
Characteristic	Median or N (%)^a^	Median or N (%)	Median or N (%)	P-value*
Median FSFI Scores Total Desire Arousal Lubrication Orgasm Satisfaction Pain	9.85 1.80 1.83 1.20 1.20 2.00 1.20	14.00 1.20 2.10 1.80 2.80 2.80 1.60	7.20 1.80 1.50 1.05 0.60 1.20 0.20	0.004 0.844 0.163 0.023 0.003 0.012 0.004
FSFI Satisfaction Score < 3.6 < 3.6 > 3.6 Unknown	76 (55) 27 (19) 36 (26)	26 (34) 16 (59) -	50 (68) 11 (41) -	0.023
Changes to Penetrative Sex (n = 66) Possible but uncomfortable Possible but painful Impossible due to pain	14 (16) 41 (62) 11 (18)	8 (57) 24 (41) 4 (36)	6 (43) 35 (59) 7 (64)	0.369
Ability to Orgasm (n = 63) Unchanged More difficult Impossible	26 (41) 26 (41) 11 (18)	6 (23) 19 (73) 6 (55)	20 (77) 7 (27) 5 (46)	< 0.001
Presenting symptoms Vaginal dryness Painful sex Low desire Hot flashes	57 (55) 46 (45) 37 (36) 23 (22)			
Genitourinary exam findings (n = 139)^b^ Vaginal dryness Loss of elasticity Loss of vascularity/pallor Loss of vaginal rugae Vaginal agglutination Vaginal scarring/adhesions Vaginal shortening Vaginal stenosis (presence of agglutination, scarring/adhesions, and/or shortening)	116 (83) 112 (81) 106 (76) 100 (74) 14 (10) 13 (9) 27 (19) 34 (23)			

* P-values are calculated either with chi-square test for association or Fisher’s exact test, excluding unknown values

^a^Some percentages do not add up to 100% due to non-response, unknown or missing values

^b^Patients could have more than one exam finding

### Vulvovaginal exam findings

**Fig. 1:**
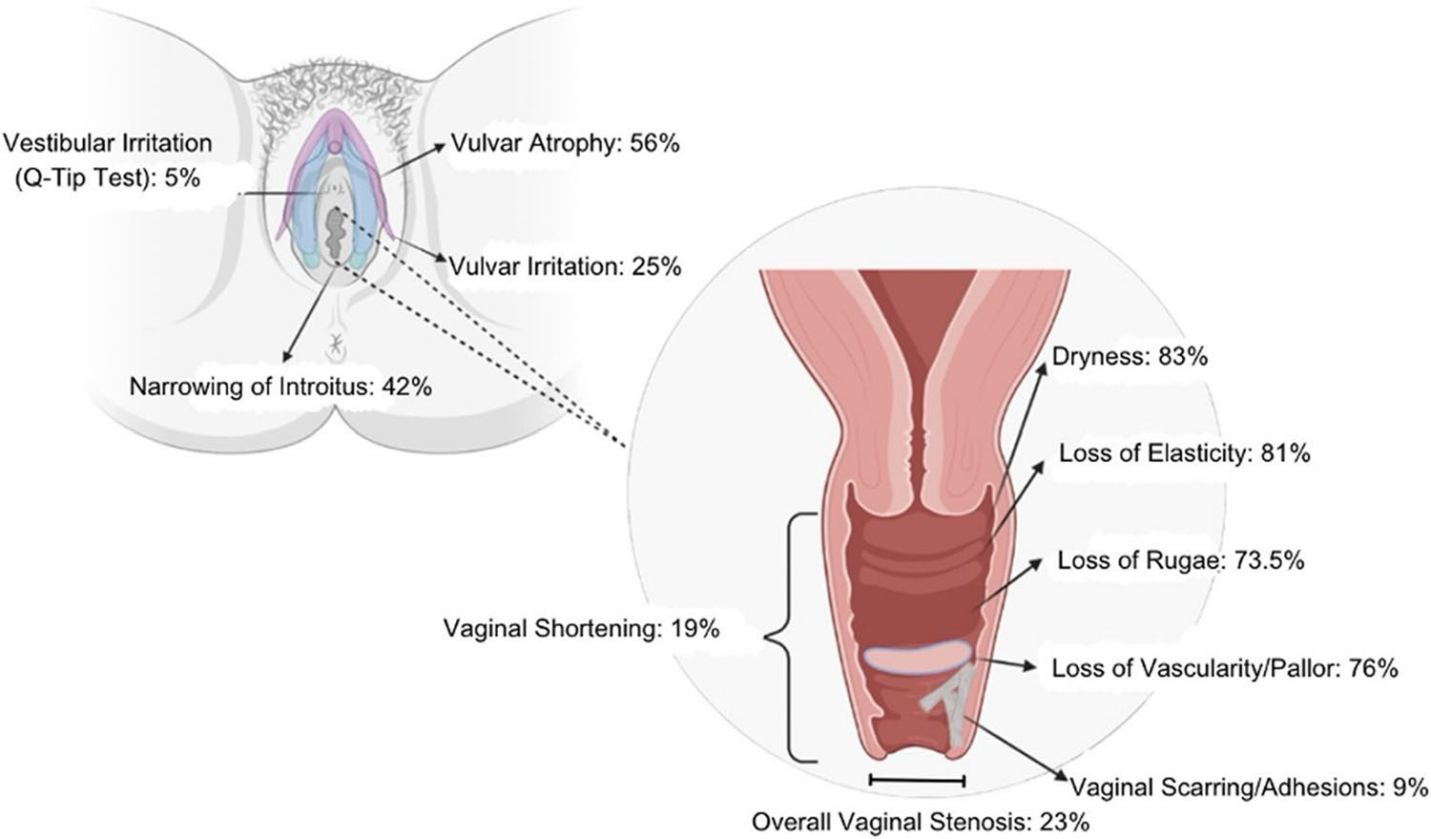
Genitourinary exam findings

Of those who underwent a GU exam (n = 139), most patients had vaginal dryness (83%), loss of elasticity (81%), loss of rugae (74%), and loss of vascularity/pallor (76%). Vaginal agglutination was observed in 10% of patients, 19% had vaginal shortening, and 9% had vaginal scarring/adhesions (Table 2, Fig. 1). Overall, the rate of vaginal stenosis in this sample was 23%. Prevalence of additional exam findings is presented in Fig. 1.

### Cancer treatment, AVES scores, and FSFI

Most patients were prescribed endocrine therapy as part of their cancer treatment (67%), including tamoxifen and aromatase inhibitors (AIs). A greater proportion of patients who were prescribed endocrine therapy were found to have AVES > 3 (p = 0.011). When analyzed by endocrine therapy type, more patients with a history of AI use had AVES > 3 (65%) compared to patients who prescribed tamoxifen only (33%) (Table 1).

**Fig. 2:**
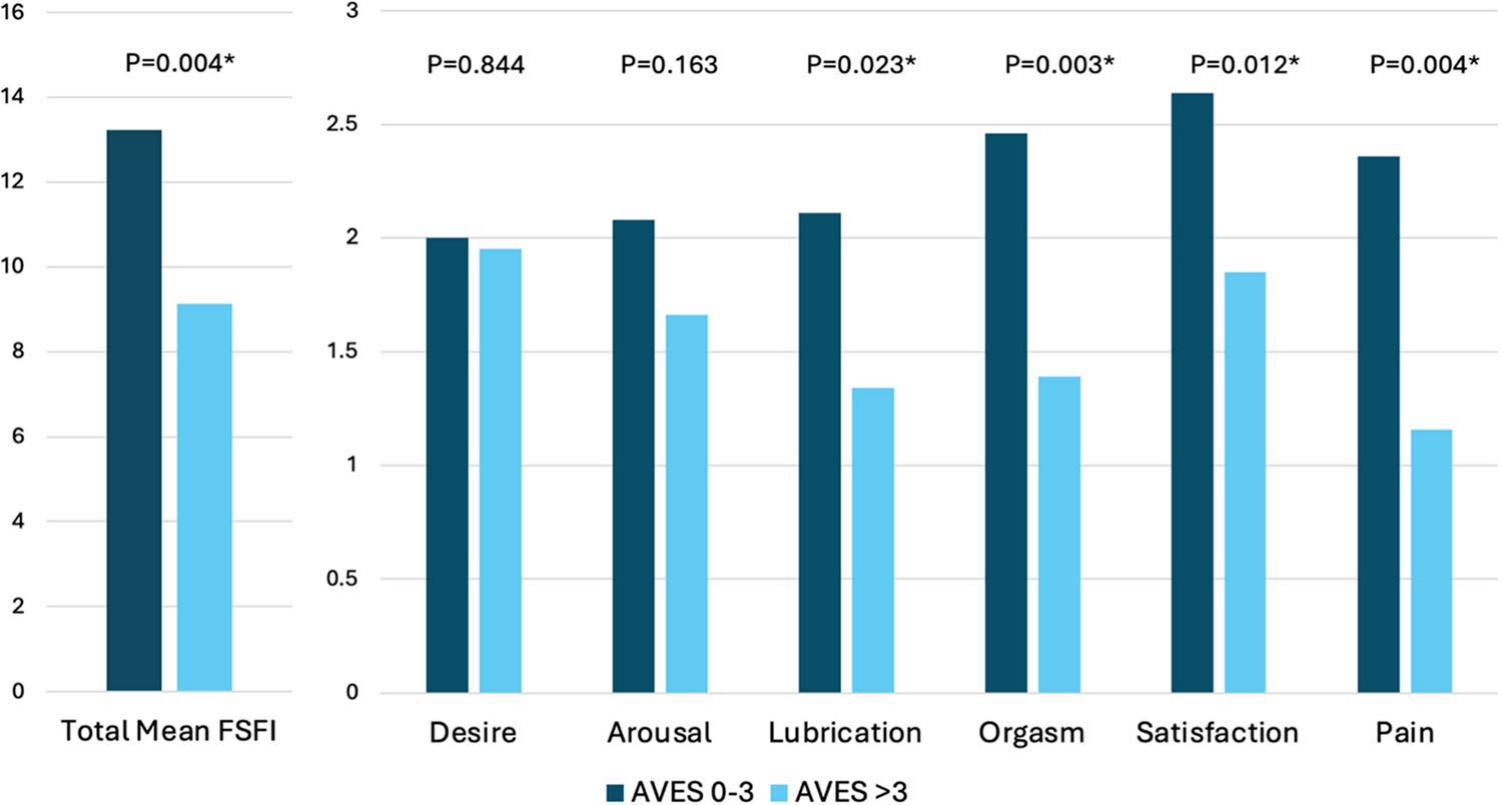
Total and domain FSFI score analyzed by AVES score groups, n = 108

108 patients completed the FSFI. Nearly every patient (n = 106, 98%) scored lower than 26.0 on the FSFI scale, suggesting a high prevalence of sexual dysfunction or lack of sexual activity. The mean FSFI score was 10.8 (SD 7.4). AVES > 3 was associated with lower FSFI scores in the lubrication, orgasm, satisfaction, and pain domains, as well as in total (Fig. 2). There were no statistically significant differences between AVES categories with respect to the FSFI desire and arousal domains. Additional analyses did not reveal an association between lower desire and arousal domain scores in patients with a history of endocrine therapy or specifically, of aromatase inhibitor use (Supplemental Table 1).

### Multivariate analysis

Multivariate analyses to determine the association of patient and treatment factors with abnormal GU exam at presentation (higher AVES) adjusted for language, smoking status, insurance, surgery, chemotherapy, radiation therapy and immunotherapy confirmed that endocrine therapy receipt was associated with higher AVES scores, as those never prescribed endocrine therapy were less likely to have severe exam disruptions (aOR 0.05, 95% CI 0.00–0.75, p = 0.030). Furthermore, FSFI domain pain scores were inversely associated with high AVES, denoting that normal (higher) pain domain score was associated with a normal GU exam (lower AVES) (aOR 0.67; 95%CI 0.48–0.95; p = 0.023) (Table 3). An additional multivariate analysis further exploring the association of FSFI satisfaction domain scores with high AVES found that women with poor FSFI satisfaction domain score (< 3.6) were nearly three times more likely to have worse AVES scores (aOR 2.81; 95%CI 1.03–7.65; p = 0.044) (Table 4).

**Table 3:** Factors associated with abnormal GU exam, where aOR < 1 is associated with normal AVES (0–3)

Covariates	Adjusted OR	95% CI	p-value
Age ≥ 50	2.62	0.39–17.52	0.320
Race (White)	1.78	0.24–13.19	0.573
Ethnicity (Non-Hispanic/Latinx)	1.95	0.42–8.94	0.391
Menopausal at Diagnosis	1.40	0.18–11.07	0.749
Any Endocrine Therapy Use FSFI Desire Domain FSFI Lubrication Domain FSFI Orgasm Domain FSFI Pain Domain	0.05 1.14 1.26 0.57 0.67	0.0–0.75 0.54–2.39 0.67–2.37 0.31–1.04 0.48–0.95	0.030* 0.733 0.475 0.066 0.023*
FSFI Satisfaction Domain	0.70	0.44–1.11	0.131
Adjusted for language, smoking status, surgery, chemotherapy, radiation therapy and immunotherapy receipt * Denotes statistically significant association

**Table 4:** Factors associated with abnormal GU exam, where aOR > 1 is associated with abnormal AVES (> 3)

Covariates	Category	Adjusted OR	95% CI	p-value
Age (years)	< 50 vs ≥ 50	0.75	0.20–2.80	0.667
Race	All Others vs White	0.60	0.14–2.64	0.503
Ethnicity	Hispanic vs Non-Hispanic	0.52	0.18–1.47	0.217
Menopause Status at Diagnosis	No vs Yes	0.53	0.13–2.19	0.380
Endocrine Therapy	Yes vs. No	0.20	0.05–0.80	0.024*
FSFI Satisfaction	< 3.6 vs 3.6 +	2.81	1.03–7.65	0.044*

*AVES* adapted vulvovaginal exam score

*FSFI* female sexual function index

AVES adapted vulvovaginal exam score

FSFI female sexual function index

## Discussion

This study is the first to correlate anatomic genitourinary exam abnormalities to patient-reported sexual function in a diverse cohort of female cancer patients. In this population, patients reported a variety of sexual side effects from treatment, including vaginal dryness, decreased sexual desire, hot flashes, and dyspareunia. Changes to orgasm and increased pain with penetrative sex were common. Higher AVES scores, corresponding to more severe genitourinary exam abnormalities, were associated with more sexual-related pain. Notably, low desire was not associated with worse AVES, suggesting that GU exam changes may not predict or explain changes to sexual desire after cancer treatment. This finding is in line with the hypothesis that sexual desire has both biological and psychological components, since the adverse effects of a cancer diagnosis and treatment may manifest in ways other than physical changes, such as emotional, mental, and psychological changes that affect sexual functioning [20]. Lastly, cancer patients with a history of endocrine therapy were more likely to have abnormal GU exams when compared to those without a history of endocrine therapy use. This finding was more pronounced in patients with a history of aromatase inhibitor use, which has not previously been characterized in a diverse population of cancer survivors.

At the inception of this study, the AVES scoring system was employed instead of the VHA as described in Eaton et. al’s study [17] because vaginal pH measurements were initially not performed during physical exams at this sexual health after cancer program. Additional components of the VHA that were not accounted for in the AVES include vaginal thickness, vulvar irritation, vulvar atrophy, vaginal irritation, vestibule irritation, incontinence, and pad use. While some of these features are reported separately in the current study, excluding these data points from the AVES limits the completeness of the analysis. However, since completion of this study, the Program has begun to utilize the vaginal health index (VHI) to quantify objective exam findings to allow for more direct data comparison across sites and with existing literature.

Elements of vaginal stenosis were identified in one-quarter of patients in this study. Previous studies characterized prevalence rates of vaginal stenosis after pelvic radiation therapy between 50 and 80% [21]. The current cohort differs from these populations because very few patients received pelvic radiation and most only received breast radiation, which highlights that iatrogenic estrogen suppression without pelvic radiation can severely disrupt vaginal anatomy. Previously, vaginal stenosis in breast cancer patients on endocrine therapy has only been reported in one study [22]. Breast medical and surgical oncologists should be aware of this complication of treatment as it chronic and progressive and can be treated when promptly identified.

Furthermore, 34% (n = 54) of the total sample had a history of hysterectomy, and half of women with vaginal stenosis had a prior hysterectomy (n = 17/34). While the current data does not specify whether the hysterectomies were due to malignancy, risk-reducing for high-risk genetic mutations, or benign indications, it is unlikely that the rate of hysterectomy in this sample significantly affected the stenosis rate. Only 9% of cancers in the total sample were gynecologic, suggesting a low rate of radical hysterectomy in which 2 cm of the vagina would be excised with the uterus for treatment of gynecologic cancer.

Elements of the female pelvic exam have been previously correlated to patient-reported sexual function outcomes. Eaton et al. found that the 4-item Vaginal Assessment Scale (VAS), a patient-reported measurement of vaginal symptoms, correlated with pain on exam [17], while Hwang et al. reported that pelvic floor muscle strength is positively associated with high desire, arousal, and satisfaction domains as well as total FSFI scores [23]. Notably, this study demonstrated a consistent inverse relationship between AVES and FSFI scores and is the first to directly correlate objective genitourinary exam abnormalities to FSFI scores.

The current study is unique in that it represents a diverse patient population with 57% Hispanic patients, identifies the relationship between FSFI score and genitourinary exam findings, and describes the prevalence of vaginal stenosis in a population of predominantly breast cancer patients. There were no significant differences identified among various demographic measures between the low and high AVES groups, but this could be attributed to sample size and the inclusion of patients with different sites of malignancy and therefore treatment protocol receipt. Additionally, while breast cancer and gynecologic cancer patients were well-represented, the population included a smaller proportion of patients with more rare cancers such as gastrointestinal and hematologic malignancies. Although the most symptomatic patients are more likely to be seen in the sexual survivorship program, which could lead to selection bias in our sample, providers should be prepared to offer targeted mitigation strategies to patients experiencing sexual function with treatment especially if a dedicated specialty program is not available.

Furthermore, patients in this study were assessed clinically after a cancer diagnosis and throughout the course of their treatment. This limits the ability to compare standardized surveys on sexual wellbeing and genitourinary exam findings before and after cancer and thereby confidently state the effects of treatment on sexual functioning. While the current study elicits some data on sexual functioning prior to cancer diagnosis (e.g. changes to penetrative sexual activity and ability to achieve orgasm), the FSFI focuses on current symptoms (within the past 4 weeks) and does not explicitly address how sexual functioning has changed compared to before a cancer diagnosis. Obtaining additional data about sexual symptoms and genitourinary exam findings prior to cancer would allow a more objective evaluation of the effects of cancer treatment on sexual functioning. Such data may identify even more significant changes to sexual functioning over time. This limitation highlights the importance of continuing to address sexual functioning and wellbeing in primary care settings, before a cancer diagnosis is identified.

While the FSFI is well-validated and commonly used to assess sexual functioning in female cancer survivors, the questionnaire does not adequately represent women who were not sexually inactive within the past four weeks. In the survey, 15 of the 19 items include a “no sexual activity” or “did not attempt intercourse” response, which is assigned a score of zero [13]. However, these questions do not distinguish whether the absence of sexual activity was due to the lowest level of sexual functioning or a lack of a sexual partner, preference to not engage in penetrative sex, or other factors unrelated to changes secondary to cancer treatment, which may impact the analysis of FSFI response data [24, 25]. The current study included all participants who returned a FSFI survey, regardless of whether the participant was currently sexually active or not. To circumvent this issue, median scores were compared instead of means in Table 1, and analyses specific to each domain including Desire and Satisfaction were included. Our ongoing work considers the number of “0” responses when analyzing total FSFI responses and employs other methods to assess sexual dysfunction that include sexually inactive women, such as the Female Sexual Distress Scale [26]. Future studies should seek to understand why women with cancer choose to not engage in sexual activity and consider this reasoning during comparative analyses.

Any practitioner who treats patients with cancer, including but not limited to breast cancer, should be aware of the potential for sexual dysfunction during and after treatment. To optimize screening and referral strategies, brief sexual symptom questionnaires can be employed in this clinical setting [27]. Best practices for discussing and treating these concerns for providers who may not be comfortable discussing these sensitive topics are described in a recent publication directed towards breast surgical oncologists who encounter symptomatic breast cancer patients on endocrine suppression [28]. Patients themselves should also be gently encouraged to have open discussions about their symptoms to bring them to the attention of their oncologic team. Failing to recognize and treat these quality-of-life concerns may impact treatment compliance and therefore oncologic outcome.

## Supplementary Information

Below is the link to the electronic supplementary material.

ESM 1 DOCX (14.2 KB)

## Data Availability

The data that support the findings of this study are stored in the University of Miami RedCap database and are available from the corresponding author upon reasonable request.
