# Supplementary material for: More than just “vaginal dryness”: sexual dysfunction correlates with genitourinary anatomy changes in female cancer survivors
Source: Support Care Cancer. 2025 Nov 13;33(12):1056. doi: 10.1007/s00520-025-10046-2 (PMC12612019; doi:10.1007/s00520-025-10046-2)
Supplement: Supplementary file 1 — DOCX (14.2 KB) [file 520_2025_10046_MOESM1_ESM.docx]

**Supplemental Table 1:** Association of any history of aromatase inhibitor therapy use on FSFI desire and arousal domain scores.

| **FSFI Scores (N=72)** | **Total**  **Mean (SD)** | **No endocrine therapy (N =34)**  **Mean (SD)** | **AI only**  **Endocrine therapy (N=38)**  **Mean (SD)** | **P-value** |
| --- | --- | --- | --- | --- |
| Desire Domain | 1.97 (1.07) | 1.92 (1.30) | 2.08 (0.83) | 0.523 |
| Arousal Domain | 1.83 (1.52) | 1.94 (1.88) | 1.83(1.51) | 0.792 |
